# Mucins trigger dispersal of *Pseudomonas aeruginosa* biofilms

**DOI:** 10.1038/s41522-018-0067-0

**Published:** 2018-10-10

**Authors:** Julia Y. Co, Gerardo Cárcamo-Oyarce, Nicole Billings, Kelsey M. Wheeler, Scott C. Grindy, Niels Holten-Andersen, Katharina Ribbeck

**Affiliations:** 1 Department of Biological Engineering, Massachusetts Institute of Technology, Cambridge, MA USA; 2 Microbiology Graduate Program, Massachusetts Institute of Technology, Cambridge, MA USA; 3 Research Laboratory of Electronics, Massachusetts Institute of Technology, Cambridge, MA USA; 4 Department of Materials Science and Engineering, Massachusetts Institute of Technology, Cambridge, MA USA

## Abstract

Mucus is a biological gel that lines all wet epithelia in the body, including the mouth, lungs, and digestive tract, and has evolved to protect the body from pathogenic infection. However, microbial pathogenesis is often studied in mucus-free environments that lack the geometric constraints and microbial interactions in physiological three-dimensional mucus gels. We developed fluid-flow and static test systems based on purified mucin polymers, the major gel-forming constituents of the mucus barrier, to understand how the mucus barrier influences bacterial virulence, particularly the integrity of *Pseudomonas aeruginosa* biofilms, which can become resistant to immune clearance and antimicrobial agents. We found that mucins separate the cells in *P*. *aeruginosa* biofilms and disperse them into suspension. Other viscous polymer solutions did not match the biofilm disruption caused by mucins, suggesting that mucin-specific properties mediate the phenomenon. Cellular dispersion depended on functional flagella, indicating a role for swimming motility. Taken together, our observations support a model in which host mucins are key players in the regulation of microbial virulence. These mucins should be considered in studies of mucosal pathogenesis and during the development of novel strategies to treat biofilms.

## Biofilms: Mucins regulate bacterial dispersal in biofilms

Biofilms are an important survival strategy for pathogenic bacteria including *Pseudomonas aeruginosa* and whilst mucins play a role the regulation of microbial virulence, microbial pathogenesis on mucosal tissues is often studied in mucin-free contexts. Here, Katharina Ribbeck and colleagues at the Massachusetts Institute of Technology used native purified mucin polymers and examined their effects on the integrity of *Pseudomonas aeruginosa* biofilms. The mucins dissolved the biofilms by separating the bacteria, which was not observed in other viscous alternative substances examined, but this did rely on functional bacterial motility. Here the authors provide evidence that mucins are involved in suppressing bacterial virulence and should be included in systems used to assess bacterial pathogenesis on mucosal tissues.

## Introduction

Human-associated bacteria often exist as biofilms, structured communities that secrete and encase themselves within a protective matrix.^1–3^ The healthy body must maintain homeostasis with these microbial communities; if growth is unchecked, then biofilms can lead to morbidities such as chronic and nosocomial infections.^4–7^ Mucus is a biological hydrogel that coats all wet epithelia in the body and forms a major ecological niche for the human microbiota, likely playing an important role in regulating host–microbe interactions.^8–11^

The gel-forming biopolymers that comprise mucus, called mucins, form bottlebrush-like structures with dense O-linked glycosylation^12,13^ and are important in maintaining health. Mucin dysregulation is associated with diseases such as cystic fibrosis,^14,15^ chronic obstructive pulmonary disorder,^16^ and ulcerative colitis,^17,18^ and can also lead to pathogen overgrowth.^14–18^ Furthermore, mucins can promote clearance of microbes.^19,20^ Mucins impair the surface attachment and formation of biofilms by *Streptococcus mutans, Candida albicans*,^21^ and *Pseudomonas aeruginosa*;^22^ surface attachment by these microbes can inflict damage on the host. Mucins also regulate the motility of *Helicobacter pylori*^23^ and suppress hyphae formation in *C. albicans*.^21^ Despite the importance of mucins in regulating pathogens, microbial physiology in these mucosal systems is often studied in mucus-free environments, which lack the biochemistry and geometric constraints found in physiological, three-dimensional mucus gels.

To close this gap, here we explored whether mucin polymers destabilize biofilms of the opportunistic pathogen *P. aeruginosa*. Biofilms of *P. aeruginosa* can become resistant to immune clearance and antimicrobial agents, threatening human health.^24–26^ We used natively purified mucins for this study because they form viscoelastic hydrogels, in contrast to the commercially available mucins that lose this ability during the purification process.^27,28^ We discovered that natively purified mucins induce the dispersal of *P. aeruginosa* biofilms, suggesting that mucins are important regulators of microbial virulence.

## Results

### Mucins disassemble *P. aeruginosa* biofilms

**Fig. 1:**
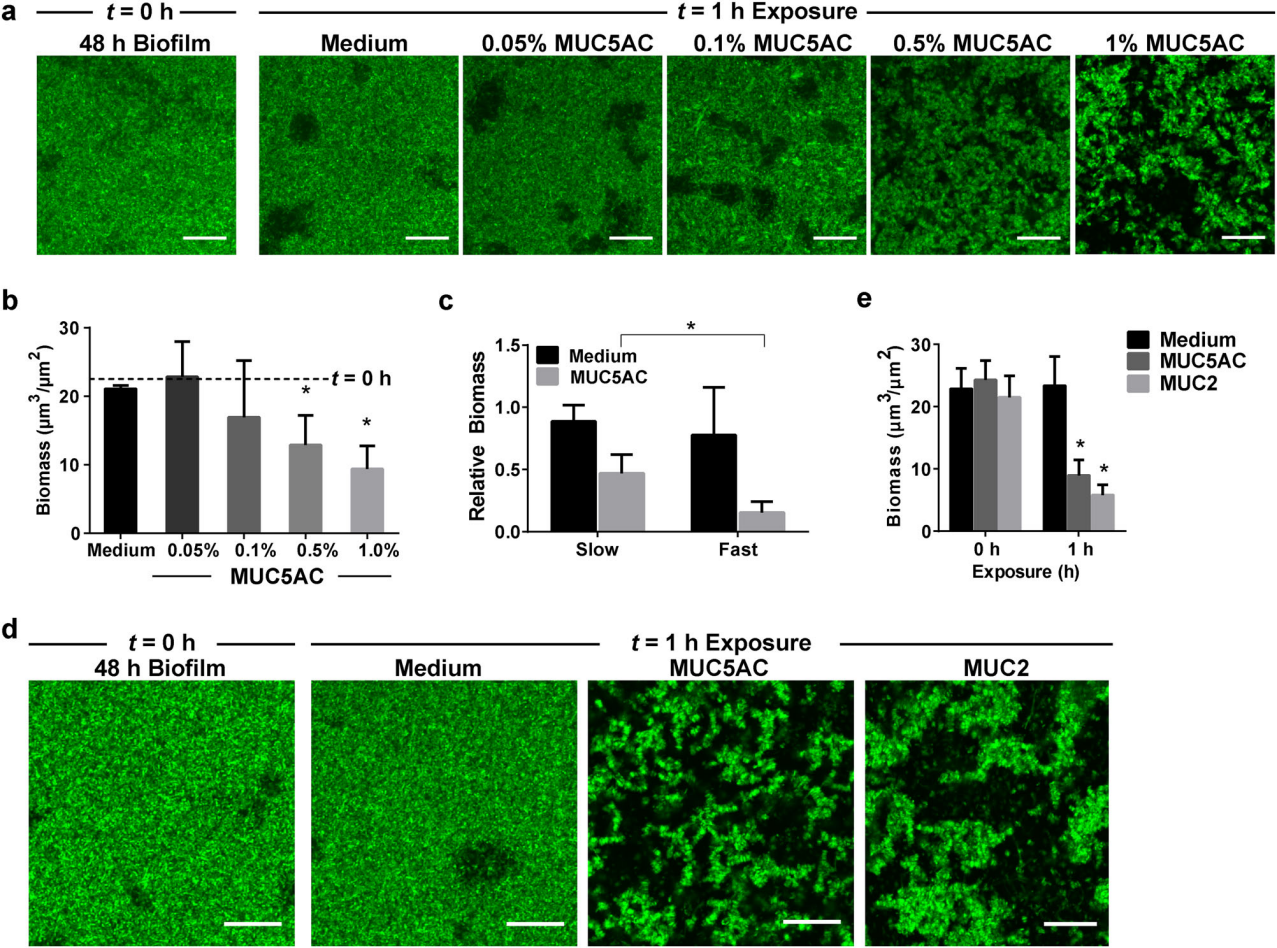
Natively purified mucins MUC5AC and MUC2 trigger the disruption of *P. aeruginosa* biofilms. **a** PAO1-GFP biofilms (48 h) were exposed to medium with increasing concentrations of mucins. The flow rate of the medium was 0.5 μL/min. At 0.5% (w/v) and above, MUC5AC reduced biofilm mass. Scale bars = 20 μm. **b** Confocal images of biofilms were analyzed using COMSTAT to quantify biomass after 1 h of exposure to mucins. Dotted lines indicate average values for 48 h biofilms before exposure (*t* = 0 h). Error bars represent standard error (*n* ≥ 3). *P ≤ 0.05, unpaired Student’s *t* test. **c** Mucin-mediated biofilm erosion is affected by the flow rate. Quantification of 48 h PAO1-GFP biofilm biomass after 1 h of exposure to LB with or without 0.5% mucins at slow flow (0.5 μL/min) or fast flow (10 μL/min). Values are normalized to biofilm biomass before exposure. Error bars represent standard error (*n* ≥ 3). *P ≤ 0.05, unpaired Student’s *t* test. A comparison between mucin MUC5AC and MUC2 shows that both mucins disassemble *P. aeruginosa* biofilms. **d** Live confocal imaging and **e** biofilm biomass quantification of 48 h PAO1-GFP biofilms before (*t* = 0 h) and after (*t* = 1 h) exposure to medium with or without 0.5% mucins at 0.5 μL/min flow. Scale bars = 20 μm. Error bars represent standard error (*n* = 3). *P ≤ 0.05, unpaired Student’s *t* test

To determine whether mucins affect the integrity of established biofilms, *P. aeruginosa* cells were grown in a previously characterized flow-cell system.^29^ This flow-cell system permits continuous replenishment of mucins, thereby preventing changes in mucin availability due to degradation or adsorption to biofilm or flow-cell surfaces. Once matured into biofilms, *P. aeruginosa* cells were exposed to mucins and the resulting effects on the biofilms were analyzed using live confocal microscopy. We used natively purified MUC5AC, the primary secreted mucin found in the lungs and stomach.^14^ Biofilms of *P. aeruginosa* PAO1 tagged with GFP (PAO1-GFP) were grown under continuous flow at 0.5 μL/min for 48 h, at which point they exhibited a relatively smooth, flat architecture (Fig. 1a). These biofilms were exposed to Luria Broth (LB) medium alone or to medium supplemented with 0.05, 0.1, 0.5, or 1.0% (w/v) mucins under continuous flow at 0.5 μL/min for 1 h, and the extent of biofilm disruption was assessed. Treatment with 0.05 and 0.1% mucins did not disassemble the biofilms, while exposure to 0.5 or 1% mucins resulted in biofilm fragmentation and disassembly (Fig. 1a, b). These data indicate that mucins effectively disrupt biofilms above a threshold mucin concentration. Based on these results, subsequent analyses were performed using mucins at a concentration of 0.5%.

Since these experiments were performed under fluid flow, the fluid mechanics (such as shear stress, which is dictated by solution viscosity and flow rate) of the system may influence biofilm growth and disassembly.^30–32^ To explore whether mucin-mediated biofilm disruption is affected by flow rate, 48 h PAO1-GFP biofilms were treated with or without 0.5% mucins for 1 h at slow flow (0.5 μL/min) or fast flow (10 μL/min). While increasing the flow of mucin-free LB did not result in substantial biofilm disassembly, fast-flow mucin treatment enhanced biofilm disassembly (Fig. 1c), resulting in biofilms with 31.6% less biomass than those treated via slow flow (Fig. 1c). Thus, a faster flow rate enhances mucin-mediated disassembly of *P. aeruginosa* biofilms.

To address whether the ability to disrupt biofilms was specific to MUC5AC, we repeated the experiment with native purified MUC2 from porcine intestinal mucus. As with exposure to MUC5AC, exposure to MUC2 for 1 h notably fragmented the biofilms (Fig. 1d, e). Confocal images of the biofilms revealed that exposure to MUC5AC and MUC2 reduced the biofilm biomass by 67.6% and 75%, respectively, compared to the mucin-free condition (Fig. 1e). Thus, both gastric and intestinal mucins disassemble *P. aeruginosa* biofilms. Due to its more facile purification, all subsequent experiments were performed with MUC5AC.

### Mucins disassemble biofilms without affecting bacterial viability

**Fig. 2:**
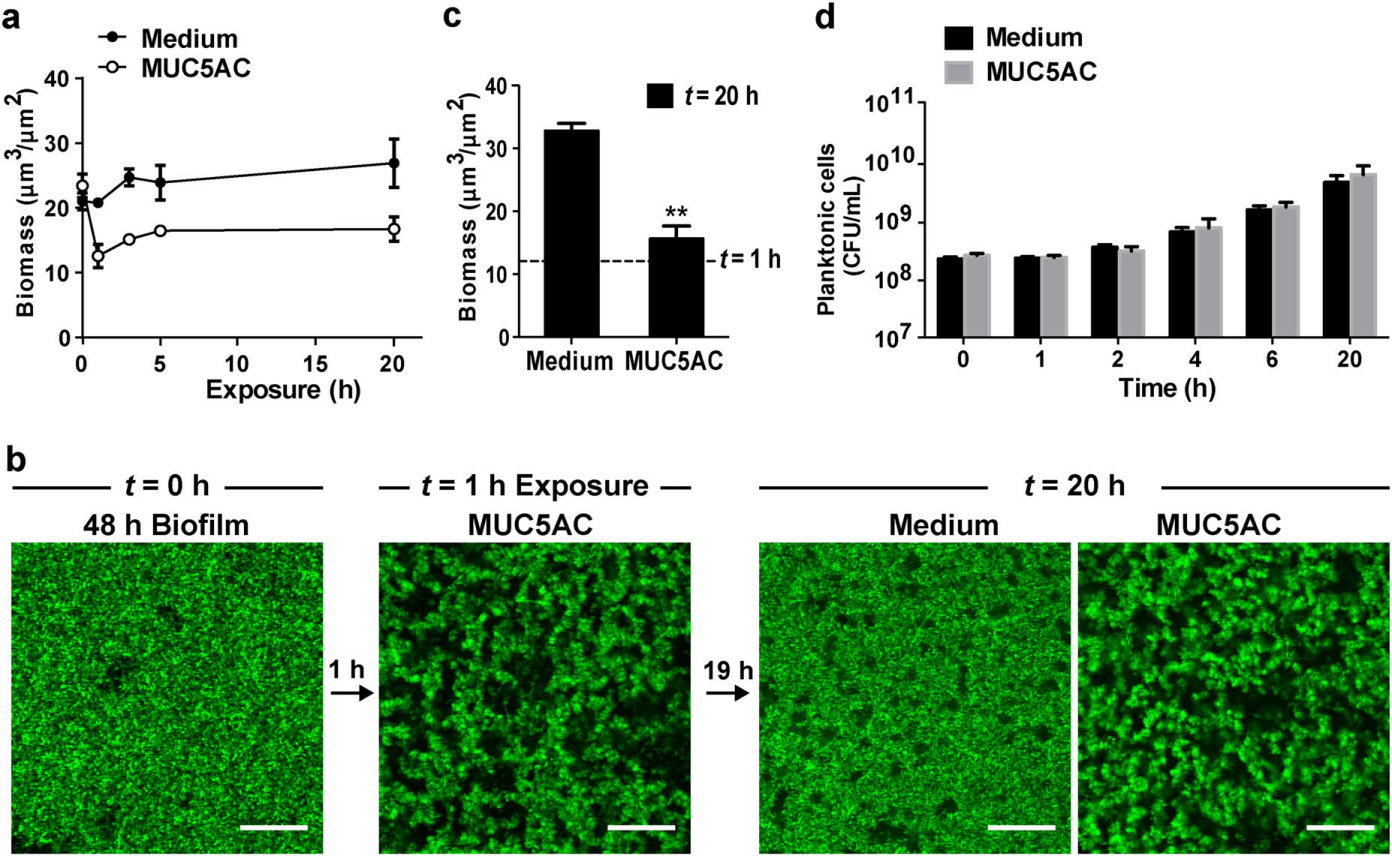
Mucins disrupt *P. aeruginosa* biofilms without killing the bacteria. **a** Prolonged mucin exposure suppresses biofilm development. 48 h PAO1-GFP biofilms were exposed to medium with or without MUC5AC (0.5% w/v) over 20 h and the remaining surface-attached biofilm biomass quantified. Error bars represent standard error (*n* = 3). **b** Biofilms eroded by exposure to mucins remain viable. 48 h PAO1-GFP biofilms were first eroded via exposure to 0.5% MUC5AC for 1 h at 0.5 μL/min flow, then incubated for an additional 19 h in medium containing MUC5AC as indicated. Biofilms resumed development after being moved to mucin-free medium, indicating that eroded biofilms are still viable. Scale bars = 20 μm. **c** Quantification of biofilm biomass at 20 h. Dotted lines indicate value after the initial 1 h mucin treatment. Error bars represent standard error (*n* ≥ 3). **P ≤ 0.005, unpaired Student’s *t* test. **d** Exposure to mucins does not significantly impair *P. aeruginosa* viability. PAO1-GFP cells were grown in suspension in medium with or without 0.5% mucins. Colony-forming units (CFUs) were counted to assess cell viability. Error bars represent standard error (*n* = 3)

While the previous experiment showed that mucins can disintegrate biofilms (Fig. 1), a residual layer of attached cells always appeared to resist dissociation from the substrate. Hence, to determine whether cells that remained in the mucin-treated biofilm could be dissociated through longer exposure to mucins, we repeated the experiment and exposed 48 h PAO1-GFP biofilms to LB medium alone or to medium supplemented with 0.5% mucins, but this time extended the exposure time to 20 h. After 20 h, the mucin-treated biofilms remained lower in biomass (Fig. 2a) than biofilms not exposed to mucin (Fig. 2a), suggesting that mucins suppress biofilm development over a relatively long period of time. However, the biofilms were not completely disintegrated, and a residual layer of biofilm cells remained (Fig. 2a).

We next determined whether this residual layer of cells could recover after mucin-mediated erosion. Biofilms were first treated with mucins for 1 h to trigger disassembly, then continually treated with mucins or allowed to recover through exposure to mucin-free medium for 19 h (Fig. 2b). Upon removal of the mucins, the biofilms began to resemble non-treated biofilms and increased in biomass nearly 3-fold relative to the biomass at 1 h (Fig. 2b). These observations suggest that mucins dissociate the outer biofilm layers, leaving behind an eroded, but viable, biofilm that can regrow. In contrast, prolonged continuous exposure to mucins maintained a fragmented biofilm structure (Fig. 2b) and resulted in only a small increase of 1.4-fold in biofilm biomass (Fig. 2c). These data corroborate our observation that mucins hinder biofilm development (Fig. 2a).

Last, we addressed whether mucins disintegrate *P. aeruginosa* biofilms by killing the bacteria. We therefore evaluated the toxicity of mucins toward *P. aeruginosa* cells. Exposure to 0.5% MUC5AC over 20 h did not affect the growth rate of planktonic PAO1-GFP cells in static culture over this period (Fig. 2d), indicating that the viability of the planktonic cells was not reduced. The absence of bactericidal or bacteriostatic effects in planktonic cells suggests that mucins are generally non-toxic to the bacteria.

### Biofilm disassembly is dependent on mucin-specific biochemistry

**Fig. 3:**
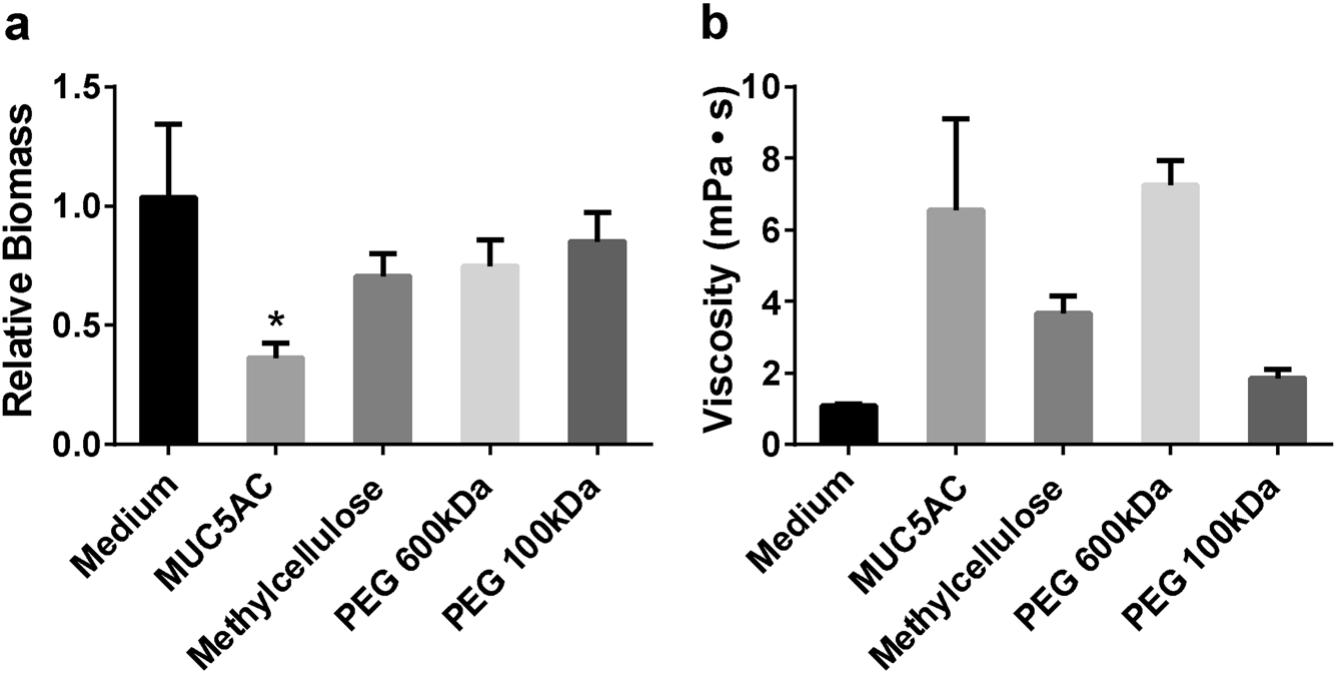
Viscous polymer solutions do not disrupt *P. aeruginosa* biofilms to the same extent as mucins. **a** Quantification of PAO1-GFP biofilms biomass (48 h) after exposure to 0.5% (w/v) polymer solutions in LB medium at 0.5 μL/min flow for 1 h shows that only mucins induce significant biofilm disassembly. Reported values represent biofilms after 1 h of exposure normalized to biofilms before exposure. Error bars represent standard error (*n* = 3). *P ≤ 0.05 versus medium-only treatment, one-way ANOVA. The medium-only treatment and non-mucin polymer treatments did not significantly differ from each other. **b** Viscosity of polymers (0.5% (w/v) solutions, *n* = 3) is not associated with the ability to disrupt *P. aeruginosa* biofilms

To investigate the specificity of mucin-mediated biofilm disassembly, we assessed the ability of several commonly studied synthetic polymer solutions to disrupt biofilms. Since mucins are heavily glycosylated polymers,^14,33^ we compared them to glycan-based polymers like methylcellulose, which has been studied as a mucin mimetic due to its similar viscoelastic properties,^34,35^ and non-glycan polymers like polyethylene glycol (PEG), which is commonly used in antifouling coatings.^36^ PAO1-GFP biofilms exposed to 0.5% methylcellulose or 0.5% PEG (600 or 100 kDa) for 1 h did not exhibit substantial biofilm disassembly (Fig. 3a). Methylcellulose treatment caused a 29.4% reduction in biomass, and 600 and 100 kDa PEG treatments resulted in similar outcomes (Fig. 3a). These data indicate that while biofilms are disrupted by other polymers, mucins exert a greater effect.

Because the viscosity of polymer solutions contributes to biofilm-disrupting shear stress,^30–32^ the rheological properties of the 0.5% polymer solutions were characterized. Mucin and 600 kDa PEG solutions had the highest viscosities, followed by methylcellulose and 100 kDa PEG (Fig. 3b). Thus, since the mucins and the 600 kDa PEG solutions had similar viscosities, but mucins more effectively disassembled biofilms, the viscosity of the polymer solutions is not directly associated with the extent of biofilm disruption. Since neither the glycan-based nor non-glycan synthetic polymers tested here disassembled the biofilms to the same degree as mucins (despite similarities in viscosity; Fig. 3b), unique biochemical or molecular properties of mucins may underlie the disruption of *P. aeruginosa* biofilms.

### Mucin-mediated biofilm disruption is dependent on the *P. aeruginosa* flagellum

**Fig. 4:**
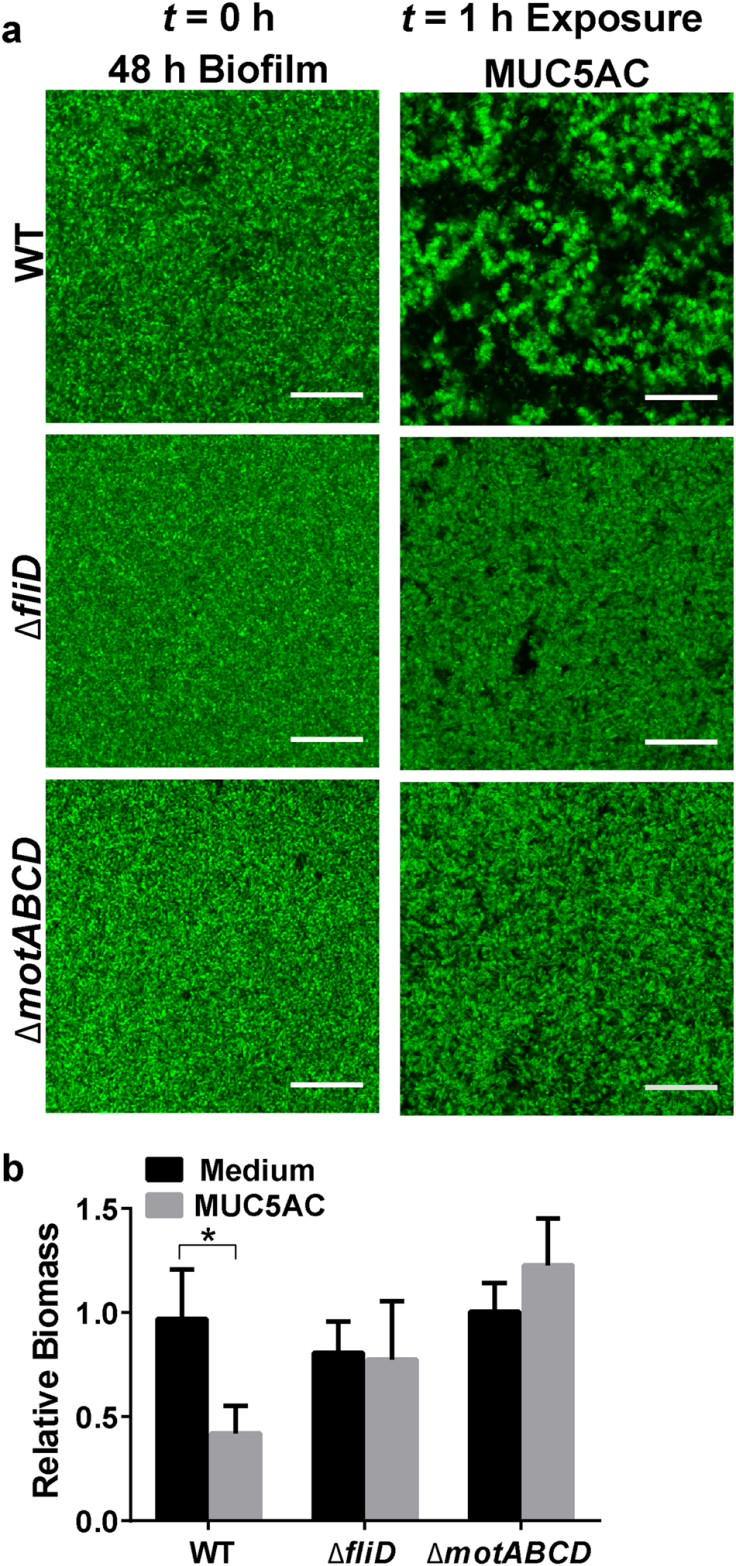
*P. aeruginosa* flagellar motility is required for mucin-associated biofilm erosion. **a** Live confocal imaging and **b** biofilm biomass quantification of PAO1-GFP (WT), PAO1Δ*fliD*-GFP, and PAO1Δ*motABCD*-GFP biofilms (48 h) exposed to LB medium with or without 0.5% mucins at 0.5 μL/min flow for 1 h. Scale bars = 20 μm. Biofilm biomass was quantified using COMSTAT analysis of confocal images. Reported values represent biofilms after 1 h of exposure normalized to WT biofilms before exposure (*t* = 0 h). Error bars represent standard error (*n* ≥ 3). *P ≤ 0.05, unpaired Student’s *t* test

Previous studies have shown that flagella, the motility appendages required for bacterial swimming through the liquid, play an important role in *P. aeruginosa* interactions with mucins.^37–39^ In addition, flagella are involved in bacterial dispersal from biofilms^40,41^ and thus may contribute to mucin-associated biofilm disruption. We therefore studied biofilms of *P. aeruginosa* lacking the flagellar cap FliD or flagellar stators MotABCD; these mutants displayed impaired swimming motility (Figure S1). When PAO1Δ*fliD*-GFP biofilms were grown in flow cells for 48 h, they closely resembled 48 h PAO1 wild-type (WT) biofilms (Fig. 4a). In contrast to WT biofilms, Δ*fliD* biofilms did not exhibit a substantial reduction in biomass after treatment with 0.5% mucins (Fig. 4b). The resistance of *fliD* mutant biofilms to mucin-mediated disruption suggests the involvement of FliD in the phenomenon.

We next examined biofilms of swimming-impaired Δ*motABCD* bacteria, which produce intact, but paralyzed, flagella.^42^ PAO1Δ*motABCD*-GFP biofilms grown for 48 h in flow cells resembled WT biofilms (Fig. 4a). Similar to the Δ*fliD* biofilms, Δ*motABCD* biofilms treated with 0.5% MUC5AC for 1 h did not disassemble (Fig. 4a, b). Thus, flagellar motility is involved in the biofilm response to mucins. Mucins may stimulate active biofilm dispersal, which in *P. aeruginosa* involves swimming motility and thus functional flagella.^40,41^

### Mucins trigger active cellular escape from biofilms

**Fig. 5:**
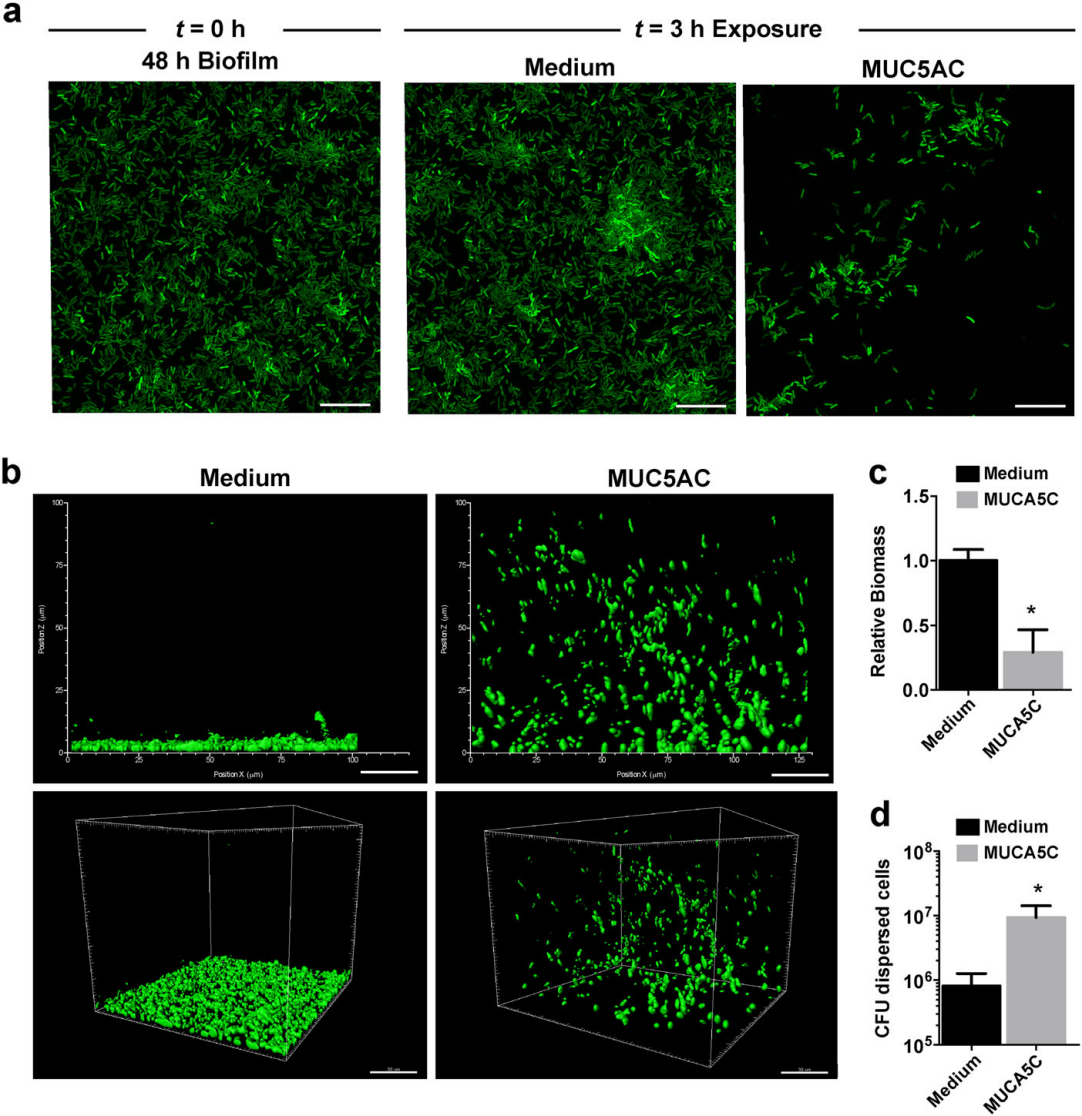
Exposure to mucins induces dispersal of *P. aeruginosa* biofilms under static growth conditions. **a** Mucins prompt active biofilm dispersal in *P. aeruginosa*. Live confocal imaging of 48 h PAO1-GFP biofilms before (*t* = 0 h) and after (*t* = 3 h) exposure to ABTG minimal medium or ABTG medium + 0.5% (w/v) MUC5AC. Scale bars = 20 μm. **b** Dispersed cells display a distinctive spatial organization. 3D representation of the cellular distribution of dispersed 48 h PAO1-GFP biofilms after 3 h of exposure to ABTG medium or to ABTG medium + 0.5% mucins. **c** Exposure to mucins reduces biomass of *P. aeruginosa* biofilms. Values are normalized to biofilm biomass before exposure. Error bars represent standard error (*n* ≥ 3). *P ≤ 0.05, unpaired Student’s *t* test. **d** Exposure to mucins increases the number of dispersed cells from *P. aeruginosa* biofilms. Viable dispersed cells were quantified by counting colony-forming units (CFUs). Error bars represent standard error (*n* ≥ 3)

If mucins induce a biofilm-escape mechanism that relies on flagellar motility, then mucin-mediated biofilm disintegration should also occur in the absence of fluid flow. To test this hypothesis, we cultured biofilms in static conditions for 48 h in glass-bottom 96-well plates and exposed them to medium containing 0.5% MUC5AC for 3 h. Confocal imaging revealed that biofilms exposed to mucin-free medium remained unaltered (Fig. 5a, c). In contrast, exposure to mucins reduced the surface-attached biofilm biomass by 70.8% (Fig. 5a, c). At the same time, after exposure to mucins, cellular aggregates of bacteria had lifted off the surface and were detected in the volume of the medium above the biofilm (Fig. 5b). These dispersed aggregates contained viable bacteria and contained a 10-fold higher cell count than the medium above untreated biofilms (Fig. 5d). This mucin-mediated disintegration of biofilms was not observed with non-motile Δ*fliD* cells (Figure S2). Although natively purified mucins are obtained through a relatively mild purification procedure to preserve their structure, it is possible that mucin-associated factors contribute to biofilm disintegration. To exclude this possibility, we tested mucins purified with cesium chloride, which removes the majority of associated proteins and lipids.^43,44^ Cesium chloride-purified mucins reduced *P. aeruginosa* biofilm biomass to a similar degree as natively purified mucins (Figure S3), indicating that this effect is likely due to the mucins and not to associated factors. These data suggest that mucins trigger an active bacterial biofilm escape, a key characteristic of biofilm dispersal.^41,45,46^

## Discussion

The mucosal barrier plays a critical role in maintaining homeostasis with host-associated biofilms. Here, we report that mucin polymers induce the disassembly of *P. aeruginosa* biofilms. Specifically, exposure to mucins resulted in disassembly of the outer layers of the biofilm, leaving behind a viable, yet eroded, structure. Mucin-induced biofilm dispersion depended on the flagellar cap encoded by *fliD* and flagellar stators encoded by *motABCD*, suggesting that flagellar motility is one component of the disassembly mechanism.

There are several mechanisms by which mucins may act upon *P. aeruginosa* biofilms. First, mucins could directly or indirectly influence bacterial signaling. For example, *P. aeruginosa* directly responds to N-acetylglucosamine, a glycan found on mucins, via a two-component regulator.^47^ Mucins may also trigger signaling events indirectly by altering nutrient availability and environmental conditions.^41,46,48^ Moreover, mucin-associated glycans may interact with specific *P. aeruginosa* surface adhesins involved in biofilm dispersion.^49^ Last, heavily glycosylated host mucins may compete against native *P. aeruginosa* polysaccharides for binding sites on the bacterial surface and within the biofilm matrix, thereby disrupting primary interactions that underlie biofilm development. A growing body of evidence points to the disruptive nature of foreign polysaccharides on the development and organization of bacterial biofilms.^50–53^

Here, we identified the flagellum as a key factor in the response of *P. aeruginosa* biofilms to mucins, consistent with previous reports that flagellar structural proteins in *P. aeruginosa* interact directly with mucins.^39,54,55^ It is possible that upon binding to mucins, the flagellum triggers intracellular signaling pathways. The importance of flagellar motility in mucin-induced biofilm disintegration is also consistent with our previous observation that mucins suppress the aggregation of *P. aeruginosa* cells into biofilm-like flocs by retaining them in their free-swimming planktonic state.^22^ Both the suppression of cellular aggregation and the disassembly of established biofilms observed here require active flagellar motility and are likely mediated by similar signaling pathways.

While exposure to mucins substantially eroded biofilms in both flow and static systems, complete biofilm eradication was not achieved. Biofilm regions that remained intact after mucin treatment may differ physiologically^56,57^ from those that are eroded; alternatively, subpopulations within the biofilm may vary in their ability to sense or respond to mucins.

Our work highlights the potential of mucins, at physiological concentrations,^58–60^ to disrupt biofilms of the problematic opportunistic pathogen *P. aeruginosa*, and points to the importance of including these polymers in experimental models of mucosal microbial pathogenesis. Can mucins impact biofilms formed by other microorganisms, or sensitize biofilms to certain antibiotics? A better understanding of the extent and mechanisms of mucin–biofilm interactions may elucidate how mucus impacts bacterial behavior in mucosal environments *in vivo*. From a therapeutic perspective, the current work indicates that native purified mucins may be leveraged to improve the efficacy of biofilm-eradicating treatments. One important challenge now is to understand the specific changes in abnormal mucins and mucus that allow biofilm formation in chronic diseases such as cystic fibrosis.^14,15^ Matsui et al.^61^ reported that both cystic fibrosis mucus and dehydrated healthy mucus, but not fully hydrated healthy mucus, promote the formation of dense colonies of *P. aeruginosa*. While the dehydrated mucus of the cystic-fibrosis lung appears to support biofilm formation of *P. aeruginosa*, the specific attributes distinguishing diseased mucus from healthy mucus have yet to be elucidated. Systematic studies comparing mucus in health and disease will be fundamental to identifying specific properties and components of mucus and mucins that confer anti-biofilm effects. Such work will provide a foundation for novel mucin-inspired strategies aimed at preventing biofilm-related disease and restoring diseased mucus to a healthy state.

## Materials and methods

### Ethics statement

The use of porcine tissues for mucin purification in this study was approved by the MIT IACUC Committee on Animal Care (CAC protocol number E13-07-0416). All protocols conformed to the USDA Animal Welfare Act and the NIH Public Health Service Policy on Humane Care and Use of Laboratory Animals.

### Bacterial strains and culture conditions

*P. aeruginosa* WT strain PAO1 and its derivatives PAO1Δ*fliD* and PAO1Δ*motABCD* have been previously described.^22^ For constitutive GFP expression, strains were transformed with pBBR1(MCS5)-Plac-*gfp*.^62^ Standard electroporation methods were employed to produce PAO1-GFP, PAO1Δ*fliD*-GFP, and PAO1Δ*motABCD*-GFP strains.

*P. aeruginosa* strains were grown in LB (Difco) with 30 μg/mL gentamicin to maintain the plasmid. For determination of planktonic cell viability, bacterial cultures were serially diluted in phosphate-buffered saline and plated on LB agar for CFU counts. OD_600_ of 0.0025 represents a culture density of ~5.0 × 10^5^ CFU/mL.

### Mucin purification

This study used native porcine gastric mucins, which differ from industrially purified mucins in their rheological properties.^27^ Native mucins were purified as previously reported.^63^ Briefly, mucus was scraped from fresh pig stomachs (Research 87, Inc., Boylston, MA, USA) and solubilized in NaCl buffer with protease inhibitors and sodium azide. Insoluble material was pelleted via ultracentrifugation and mucins were purified using size-exclusion chromatography on a Sepharose CL-2B column (GE Healthcare Life Sciences). Mucin fractions were desalted, concentrated, and lyophilized for storage. Intestinal mucins were isolated from the mucus of fresh porcine small intestines and purified as described above for native gastric mucins. To ensure that there were no contaminants in the mucin preparation, mucins prepared via CsCl gradient centrifugation as previously reported^43,44^ were compared to those prepared without this step.

### Flow-cell biofilm reactor

Biofilms were grown in flow cells supplied with LB medium. The flow system was assembled and prepared as described previously.^62^ Briefly, a polydimethylsiloxane (Sylgard 184; Dow Corning) microfluidic device was molded from capillaries anchored to a petri dish, yielding a negative imprint of four straight microchannels (4 × 2 × 35 mm); this imprint was bonded to a glass coverslip. A suspension of cells at OD_600_ = 0.0025 was introduced into the microchannels under continuous flow (0.5 μL/min unless indicated otherwise) driven by a syringe pump (PHD Ultra, Harvard Apparatus, Holliston MA, USA) for 48 h at room temperature. Fresh medium was introduced for 3 min at 25 μL/min before treatment or analysis to remove the unattached cells from the biofilm surface, allowing for clearer detection of the attached cells by confocal microscopy. This wash step is included in control treatments (mucin-free media) and does not affect biofilm morphology. Mucins, methylcellulose (15 cP Sigma-Aldrich), PEG 100 kDa (Sigma-Aldrich), or PEG 600 kDa (Sigma-Aldrich) in LB were introduced into channels at 0.5% (w/v), unless otherwise stated, for the indicated treatment times. Biofilms were imaged before and after treatments at the middle of the channel using a Zeiss 510 confocal laser-scanning microscope with a 20×/0.5 NA dry objective with 2× or 4× zoom or a 100×/1.4 NA oil immersion objective for high-magnification images. The excitation wavelength for GFP was 488 nm. One stack was recorded for each flow cell and at least three independent flow cells were analyzed for each condition. Images were acquired with a step size of 0.5 μm. Fluorescence signal from GFP-expressing bacteria was quantified using COMSTAT^64^ to determine biofilm biomass. Biofilm biomass after treatments is reported as absolute values (μm^3^/μm^2^) or as relative biomass (normalized to biofilm biomass before treatments).

### Rheology

Rheological tests were performed on an MCR 302 rheometer (Anton-Paar) in a cone-plate geometry. The diameter of the cone was 25 mm, the cone angle was 1°, and the cone truncation was 51 μm. Ninety microliters of 0.5% polymer solutions solubilized in LB were applied to the rheometer. Shear stress was measured at shear rates of *γ̇* = 10/s to 100/s. Viscosity was calculated assuming a Newtonian relationship between the stress and the shear rate, *τ*(*γ̇*) = *ηγ̇*.

### Biofilm dispersal under static conditions

*P. aeruginosa* biofilm dispersal was assayed as previously described^65^ with the following modifications. Briefly, an overnight culture of PAO1-GFP was prepared in LB with shaking at 37 °C. Overnight cultures were diluted in ABTG medium to an initial OD_600_ of 0.01, added to a glass-bottom 96-well plate, and incubated for 48 h at 37 °C. The supernatant containing non-adherent cells was removed from the plate and the biofilm remaining in each well was washed at least three times with 0.9% NaCl. Biofilms were exposed to ABTG medium alone or ABTG medium + 0.5% mucins (MUC5AC) and incubated at 37 °C with gentle shaking (<80 rpm) for 3 h. Plates were either examined via microscopy to determine the cellular distribution of dispersed cells in each well or the biofilms in all wells were washed three times with 0.9% NaCl and resuspended in ABTG medium for microscopy to quantify the remaining biofilm biomass. Viable dispersed cells were quantified via CFU counts on LB agar plates. Experiments were performed in triplicate.

Image acquisition was performed using a confocal laser scanning microscope (LSM 800; Zeiss) equipped with a 63×/1.4 NA oil immersion or a 100×/1.4 NA oil immersion objective. Images were analyzed with Zeiss ZEN 2.1 imaging software (Thornwood, NY, USA). The excitation wavelength for GFP was 488 nm. At least five stacks were recorded for each well and at least three independent wells were analyzed for each condition. Images were acquired with a step size of 0.5 μm. Biofilm quantification was performed using COMSTAT 1.^66^ 3D images of biofilms and planktonic cells (Fig. 5b) were created with IMARIS 7.7.2 (Bitplane, Switzerland).

### Swimming motility

Swimming motility was evaluated using a previously described plate-based assay.^67^ *P. aeruginosa* strains were grown overnight on LB agar plates at 37 °C. Using a sterile pipette tip, colonies were picked and stabbed into swimming agar plates (M8 salts, 1 mM MgSO_4_, 0.2% glucose, 0.5% casamino acids, 0.3% agar). Plates were incubated upright for 24 h at room temperature, and diameters of the swim zones were measured.

### Statistical analysis

Analyses were performed using PRISM (GraphPad Software). To determine the statistical significance of the difference between the means for two experimental groups, an unpaired, two-tailed Student’s *t* test was used. For the polymer-treatment experiment in which multiple experimental conditions were compared to the LB control, one-way ANOVA with Dunnett’s multiple comparison test was used. Differences were considered statistically significant if *P* ≤ 0.05.

## Electronic supplementary material

Supplemental Information

## Data Availability

The authors declare that all relevant data supporting the findings of the study are available in this article and its Supplementary Information files, or from the corresponding author upon request.

## Electronic supplementary material

**Supplementary information** accompanies the paper on the *npj Biofilms and Microbiomes* website (10.1038/s41522-018-0067-0).

## References

[1] O’Toole G, Kaplan HB, Kolter R (2000). Biofilm formation as microbial development. Annu. Rev. Microbiol..

[2] Hall-Stoodley L, Costerton JW, Stoodley P (2004). Bacterial biofilms: from the natural environment to infectious diseases. Nat. Rev. Microbiol..

[3] Flemming HC, Neu TR, Wozniak DJ (2007). The EPS matrix: the ‘house of biofilm cells’. J. Bacteriol..

[4] Darouiche RO (2001). Device-associated infections: a macroproblem that starts with microadherence. Clin. Infect. Dis..

[5] Wozniak DJ (2003). Alginate is not a significant component of the extracellular polysaccharide matrix of PA14 and PAO1 *Pseudomonas aeruginosa* biofilms. Proc. Natl. Acad. Sci. U.S.A..

[6] Hall-Stoodley L, Stoodley P (2005). Biofilm formation and dispersal and the transmission of human pathogens. Trends Microbiol..

[7] Magill SS (2014). Multistate point-prevalence survey of health care-associated infections. N. Engl. J. Med..

[8] Johansson ME (2008). The inner of the two Muc2 mucin-dependent mucus layers in colon is devoid of bacteria. Proc. Natl. Acad. Sci. U.S.A..

[9] Linden SK, Sutton P, Karlsson NG, Korolik V, McGuckin MA (2008). Mucins in the mucosal barrier to infection. Mucosal Immunol..

[10] Derrien M (2010). Mucin–bacterial interactions in the human oral cavity and digestive tract. Gut Microbes.

[11] McGuckin MA, Lindén SK, Sutton P, Florin TH (2011). Mucin dynamics and enteric pathogens. Nat. Rev. Microbiol..

[12] Bansil R, Turner BS (2006). Mucin structure, aggregation, physiological functions and biomedical applications. Curr. Opin. Colloid Interface Sci..

[13] Hattrup CL, Gendler SJ (2008). Structure and function of the cell surface (tethered) mucins. Annu. Rev. Physiol..

[14] Rose MC, Voynow JA (2006). Respiratory tract mucin genes and mucin glycoproteins in health and disease. Physiol. Rev..

[15] Henke MO, John G, Germann M, Lindemann H, Rubin BK (2007). MUC5AC and MUC5B mucins increase in cystic fibrosis airway secretions during pulmonary exacerbation. Am. J. Respir. Crit. Care Med..

[16] Caramori G (2004). Mucin expression in peripheral airways of patients with chronic obstructive pulmonary disease. Histopathology.

[17] Van der Sluis M (2006). Muc2-deficient mice spontaneously develop colitis, indicating that MUC2 is critical for colonic protection. Gastroenterology.

[18] Heazlewood CK (2008). Aberrant mucin assembly in mice causes endoplasmic reticulum stress and spontaneous inflammation resembling ulcerative colitis. PLoS Med..

[19] Tilley AE, Walters MS, Shaykhiev R, Crystal RG (2015). Cilia dysfunction in lung disease. Annu. Rev. Physiol..

[20] Zemanick ET, Hoffman LR (2016). Cystic fibrosis: microbiology and host response. Pediatr. Clin. North Am..

[21] Kavanaugh NL, Zhang AQ, Nobile CJ, Johnson AD, Ribbeck K (2014). Mucins suppress virulence traits of *Candida albicans*. mBio.

[22] Caldara M (2012). Mucin biopolymers prevent bacterial aggregation by retaining cells in the free-swimming state. Curr. Biol..

[23] Celli JP (2009). *Helicobacter pylori* moves through mucus by reducing mucin viscoelasticity. Proc. Natl. Acad. Sci. U.S.A..

[24] Stewart PS, Costerton JW (2001). Antibiotic resistance of bacteria in biofilms. Lancet.

[25] Høiby N, Bjarnsholt T, Givskov M, Molin S, Ciofu O (2010). Antibiotic resistance of bacterial biofilms. Int. J. Antimicrob. Agents.

[26] Bjarnsholt T (2013). The in vivo biofilm. Trends Microbiol..

[27] Kocevar-Nared J, Kristl J, Smid-Korbar J (1997). Comparative rheological investigation of crude gastric mucin and natural gastric mucus. Biomaterials.

[28] Crater JS, Carrier RL (2010). Barrier properties of gastrointestinal mucus to nanoparticle transport. Macromol. Biosci..

[29] Billings N, Rusconi R, Stocker R, Ribbeck K (2014). Microfluidic-based time-kill kinetic assay. Bio-Protocol.

[30] Wanner O, Cunningham AB, Lundman R (1995). Modeling biofilm accumulation and mass transport in a porous medium under high substrate loading. Biotechnol. Bioeng..

[31] Stoodley P, Lewandowski Z, Boyle JD, Lappin-Scott HM (1999). Structural deformation of bacterial biofilms caused by short-term fluctuations in fluid shear: an in situ investigation of biofilm rheology. Biotechnol. Bioeng..

[32] Stoodley P (2001). Growth and detachment of cell clusters from mature mixed-species biofilms. Appl. Environ. Microbiol..

[33] Thornton DJ, Sheehan JK (2004). From mucins to mucus: toward a more coherent understanding of this essential barrier. Proc. Am. Thorac. Soc..

[34] Worku ML (1999). Motility of *Helicobacter pylori* in a viscous environment. Eur. J. Gastroenterol. Hepatol..

[35] Smith DJ, Gaffney EA, Gadelha H, Kapur N, Kirkman-Brown JC (2009). Bend propagation in the flagella of migrating human sperm, and its modulation by viscosity. Cell Motil. Cytoskelet..

[36] Banerjee I, Pangule RC, Kane RS (2011). Antifouling coatings: recent developments in the design of surfaces that prevent fouling by proteins, bacteria, and marine organisms. Adv. Mater..

[37] Arora SK, Ritchings BW, Almira EC, Lory S, Ramphal R (1998). The *Pseudomonas aeruginosa* flagellar cap protein, FliD, is responsible for mucin adhesion. Infect. Immun..

[38] Scharfman A (2001). Recognition of Lewis x derivatives present on mucins by flagellar components of *Pseudomonas aeruginosa*. Infect. Immun..

[39] Landry RM, An D, Hupp JT, Singh PK, Parsek MR (2006). Mucin–*Pseudomonas aeruginosa* interactions promote biofilm formation and antibiotic resistance. Mol. Microbiol..

[40] Sauer K, Camper AK, Ehrlich GD, Costerton JW, Davies DG (2002). *Pseudomonas aeruginosa* displays multiple phenotypes during development as a biofilm. J. Bacteriol..

[41] Davies, D. G. Biofilm dispersion. In *Biofilm Highlights* (eds. Flemming, H.-C., Wingender, J. & Szewzyk, U.) Vol. 5, 1–28 (Springer, Berlin, Heidelberg, 2011).

[42] Toutain CM, Zegans ME, O’Toole GA (2005). Evidence for two flagellar stators and their role in the motility of *Pseudomonas aeruginosa*. J. Bacteriol..

[43] Smith BF, LaMont JT (1984). Hydrophobic binding properties of bovine gallbladder mucin. J. Biol. Chem..

[44] Gong DH, Turner B, Bhaskar KR, Lamont JT (1990). Lipid binding to gastric mucin: protective effect against oxygen radicals. Am. J. Physiol..

[45] Chua SL (2014). Dispersed cells represent a distinct stage in the transition from bacterial biofilm to planktonic lifestyles. Nat. Commun..

[46] Petrova OE, Sauer K (2016). Escaping the biofilm in more than one way: desorption, detachment or dispersion. Curr. Opin. Microbiol..

[47] Korgaonkar A, Trivedi U, Rumbaugh KP, Whiteley M (2013). Community surveillance enhances *Pseudomonas aeruginosa* virulence during polymicrobial infection. Proc. Natl. Acad. Sci. U.S.A..

[48] McDougald D, Rice SA, Barraud N, Steinberg PD, Kjelleberg S (2011). Should we stay or should we go: mechanisms and ecological consequences for biofilm dispersal. Nat. Rev. Microbiol..

[49] Johansson EM (2008). Inhibition and dispersion of *Pseudomonas aeruginosa* biofilms by glycopeptide dendrimers targeting the fucose-specific lectin LecB. Chem. Biol..

[50] Karwacki MT (2013). Antibiofilm activity of *Actinobacillus pleuropneumoniae* serotype 5 capsular polysaccharide. PLoS One.

[51] Rendueles O, Kaplan JB, Ghigo JM (2013). Antibiofilm polysaccharides. Environ. Microbiol..

[52] Murugan K, Selvanayaki K, Al-Sohaibani S (2016). Urinary catheter indwelling clinical pathogen biofilm formation, exopolysaccharide characterization and their growth influencing parameters. Saudi J. Biol. Sci..

[53] Wu S, Liu G, Jin W, Xiu P, Sun C (2016). Antibiofilm and anti-Infection of a marine bacterial exopolysaccharide against *Pseudomonas aeruginosa*. Front. Microbiol..

[54] Simpson DA, Ramphal R, Lory S (1992). Genetic analysis of *Pseudomonas aeruginosa* adherence: distinct genetic loci control attachment to epithelial cells and mucins. Infect. Immun..

[55] Arora SK, Ritchings BW, Almira EC, Lory S, Ramphal R (1996). Cloning and characterization of *Pseudomonas aeruginosa* fliF, necessary for flagellar assembly and bacterial adherence to mucin. Infect. Immun..

[56] Tolker-Nielsen T, Molin S (2000). Spatial organization of microbial biofilm communities. Microb. Ecol..

[57] Stewart PS, Franklin MJ (2008). Physiological heterogeneity in biofilms. Nat. Rev. Microbiol..

[58] Fahy JV, Dickey BF (2010). Airway mucus function and dysfunction. N. Engl. J. Med..

[59] Adler KB, Tuvim MJ, Dickey BF (2013). Regulated mucin secretion from airway epithelial cells. Front. Endocrinol..

[60] Livraghi-Butrico A (2017). Contribution of mucus concentration and secreted mucins Muc5ac and Muc5b to the pathogenesis of muco-obstructive lung disease. Mucosal Immunol..

[61] Matsui H (2006). A physical linkage between cystic fibrosis airway surface dehydration and *Pseudomonas aeruginosa* biofilms. Proc. Natl. Acad. Sci. U.S.A..

[62] Billings N (2013). The extracellular matrix component Psl provides fast-acting antibiotic defense in *Pseudomonas aeruginosa* biofilms. PLoS Pathog..

[63] Lieleg O, Lieleg C, Bloom J, Buck CB, Ribbeck K (2012). Mucin biopolymers as broad-spectrum antiviral agents. Biomacromolecules.

[64] Heydorn A (2000). Quantification of biofilm structures by the novel computer program COMSTAT. Microbiology.

[65] Chua SL (2015). In vitro and in vivo generation and characterization of *Pseudomonas aeruginosa* biofilm-dispersed cells via c-di-GMP manipulation. Nat. Protoc..

[66] Heydorn A (2000). Experimental reproducibility in flow-chamber biofilms. Microbiology.

[67] Ha DG, Kuchma SL, O’Toole GA (2014). Plate-based assay for swimming motility in *Pseudomonas aeruginosa* in. Methods Mol. Biol..

