# Supplementary material for: Mucins trigger dispersal of Pseudomonas aeruginosa biofilms
Source: NPJ Biofilms Microbiomes. 2018 Oct 10;4:23. doi: 10.1038/s41522-018-0067-0 (PMC6180003; doi:10.1038/s41522-018-0067-0)
Supplement: Supplementary file 1 — Supplemental Information [file 41522_2018_67_MOESM1_ESM.pdf]

Supplemental Information for "Mucins trigger dispersal of *Pseudomonas aeruginosa* biofilms"

Julia Y Co[a,b*], Gerardo Cárcamo-Oyarce[c*], Nicole Billings[a], Kelsey M Wheeler[a,b], Scott C Grindy[d], Niels Holten-Andersen[d], Katharina Ribbeck[a]#

[a]Department of Biological Engineering, Massachusetts Institute of Technology, Cambridge, Massachusetts, USA; [b]Microbiology Graduate Program, Massachusetts Institute of Technology, Cambridge, Massachusetts, USA; [c]Research Laboratory of Electronics, Massachusetts Institute of Technology, Cambridge, Massachusetts, USA; [d]Department of Materials Science and Engineering, Massachusetts Institute of Technology, Cambridge, Massachusetts, USA

Supplemental Figures

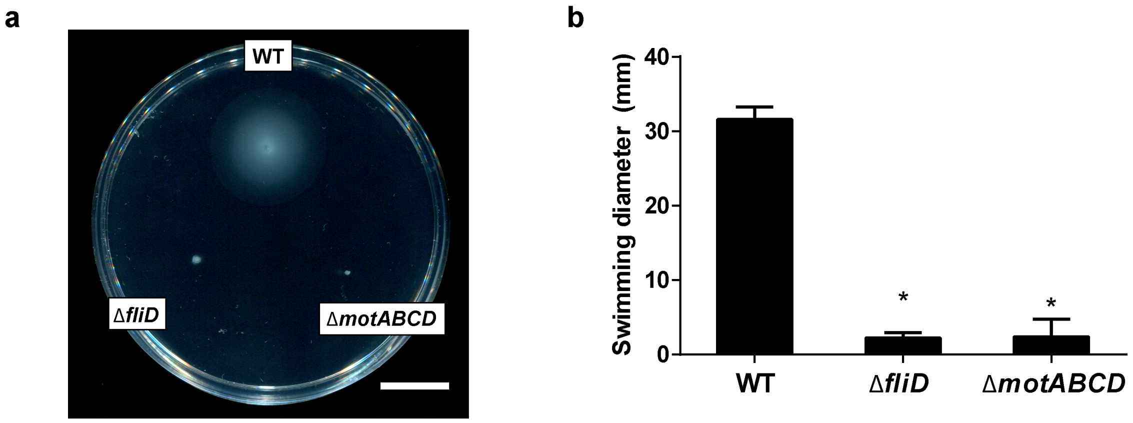

Figure S1. *P. aeruginosa* flagella-deficient mutants exhibit impaired swimming motility. (a) *P. aeruginosa* cells were inoculated onto a swim agar plate (M8 medium with 0.3% agar) and imaged after 24 h at room temperature. (b) Diameters of swim zones were measured for each strain. Error bars represent standard error ($n = 4$). *$P \leq 0.05$ versus WT, unpaired Student's t test.

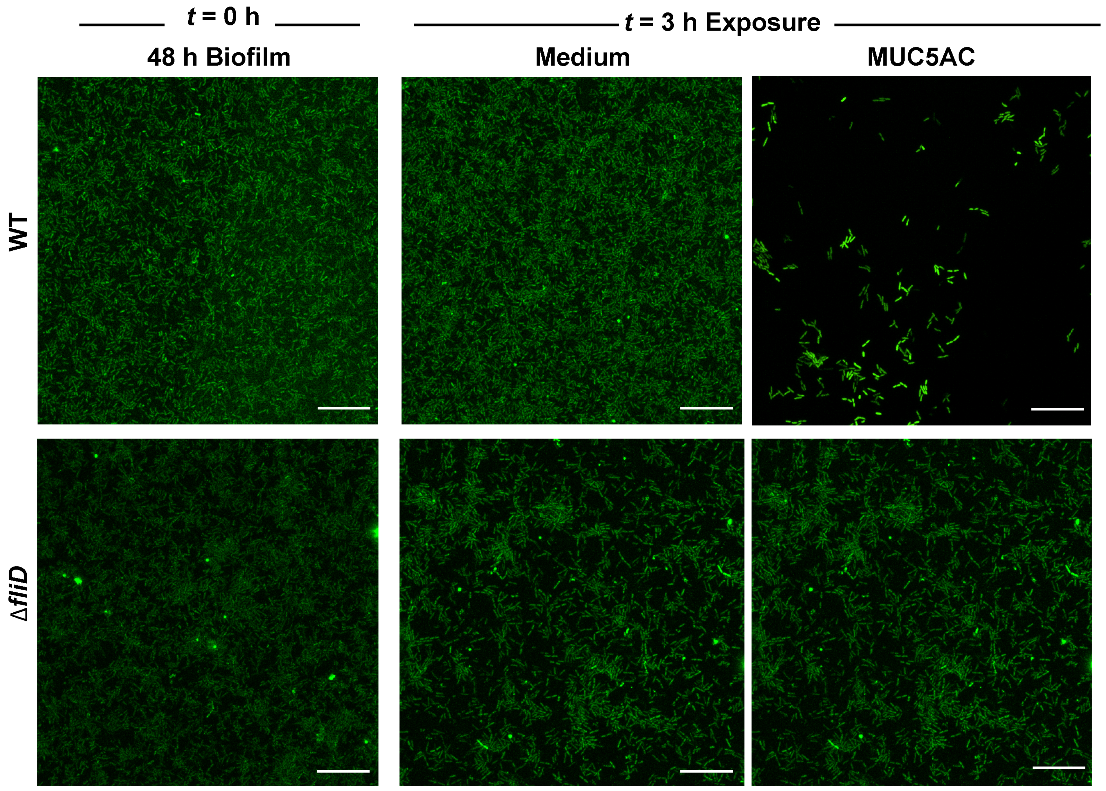

Figure S2. *P. aeruginosa* flagellar motility is required for mucin-induced biofilm dispersion. PAO1-GFP (WT) and PAO1Δ*fliD*-GFP biofilms (48 h) were exposed to ABTG medium with or without 0.5% mucins. Scale bars = 20 µm.

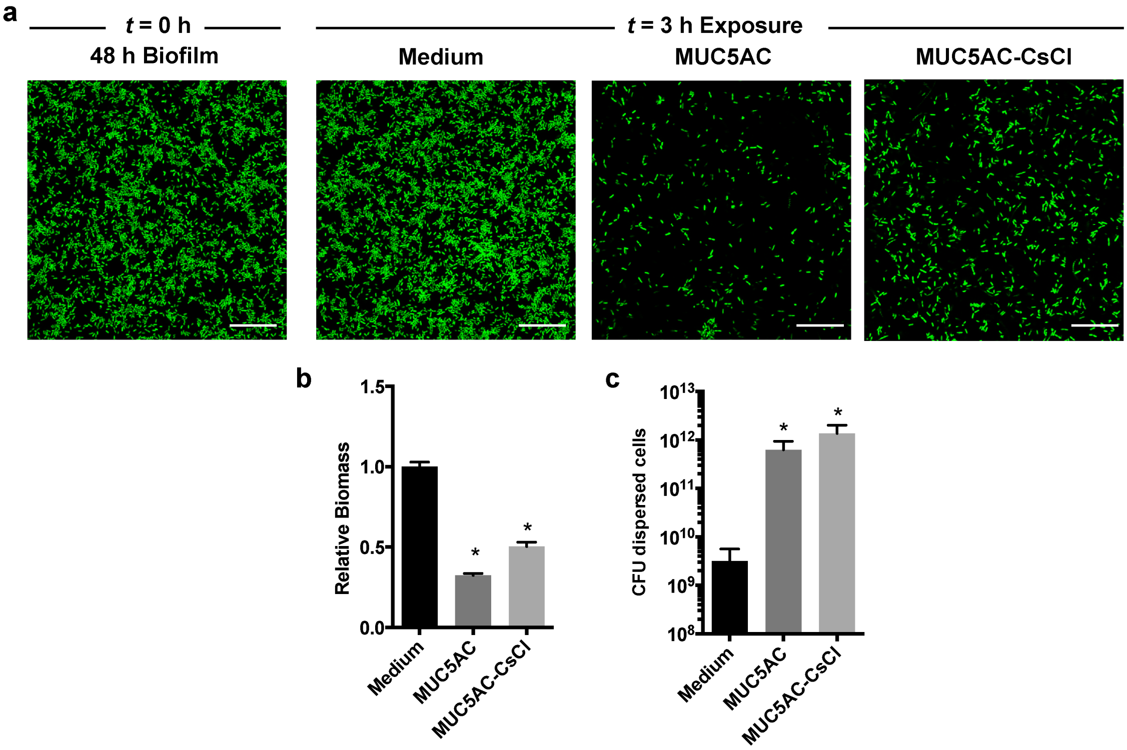

Figure S3. CsCl-purified mucin induces dispersal of *P. aeruginosa* biofilms under static growth conditions. (a) Live confocal imaging and (b) biofilm biomass quantification of 48 h PAO1-GFP biofilms before (t = 0 h) and after (t = 3 h) exposure to ABTG minimal medium, ABTG medium + 0.5% mucins, or ABTG medium + 0.5% CsCl-purified mucins. Scale bars = 20 μm. Values are normalized to biofilm biomass before exposure. Error bars represent standard error ($n \geq 3$). *$P \leq 0.05$, unpaired Student's t test. (c) Exposure to CsCl-purified mucin increases the number of dispersed cells from *P. aeruginosa* biofilms. Colony-forming units (CFUs) were counted to determine cell viability. Error bars represent standard error (n = 3).
